# Magnetothermally Responsive Mesoporous Silica Nanocarriers: Materials Design, Thermoresponsive Gates and Controlled Drug Release

**DOI:** 10.3390/nano16161018

**Published:** 2026-08-18

**Authors:** Juliana Jesus, Manuel Graça, Ana Salomé Pires, Susana Devesa, Sílvia Soreto Teixeira

**Affiliations:** 1 i3N and Department of Physics, University of Aveiro, Campus Universitário de Santiago, 3810-193 Aveiro, Portugal; julianajesus@ua.pt (J.J.); mpfg@ua.pt (M.G.); 2 Coimbra Institute for Clinical and Biomedical Research (iCBR) Area of Environment Genetics and Oncobiology (CIMAGO), Institute of Biophysics, Faculty of Medicine, University of Coimbra, Azinhaga de Santa Comba, Pólo III—Pólo das Ciências da Saúde, 3000-548 Coimbra, Portugal; pireslourenco@uc.pt; 3 Center for Innovative Biomedicine and Biotechnology (CIBB), University of Coimbra, Rua Larga, 3004-504 Coimbra, Portugal; 4 Clinical Academic Center of Coimbra (CACC), Praceta Professor Mota Pinto, 3004-561 Coimbra, Portugal; 5 Centre for Mechanical Engineering, Materials and Processes (CEMMPRE), Advanced Production and Intelligent Systems Associated Laboratory (ARISE), Department of Mechanical Engineering, University of Coimbra, Rua Luís Reis Santos, 3030-788 Coimbra, Portugal

**Keywords:** magnetic hyperthermia, Fe_3_O_4_@mSiO_2_, thermoresponsive polymers, controlled drug release, nanomedicine translation

## Abstract

Magnetothermally responsive nanocarriers represent a promising platform for spatio-temporally controlled drug delivery by combining alternating magnetic field (AMF)-induced heating with thermally triggered cargo release. Among the available architectures, magnetite-core/mesoporous-silica-shell (Fe_3_O_4_@mSiO_2_) nanoparticles functionalized with thermoresponsive polymer gatekeepers are particularly attractive. These systems integrate a magnetic heat source, a mesoporous drug reservoir, and temperature-dependent control of pore accessibility. This review examines the fundamental principles of magnetic hyperthermia, including heat-generation mechanisms, specific absorption rate (SAR), intrinsic loss power (ILP), AMF parameters and safety, and the interplay between Néel and Brownian relaxation. It also critically discusses core–shell synthesis and architecture, drug-loading strategies, PNIPAM-, PNVCL-, and other LCST-type gatekeepers, and the physicochemical characterization required to validate the complete nanocarrier. Evidence for combined magnetic hyperthermia and chemotherapy is assessed together with hemocompatibility, immunogenicity, oxidative stress, biodistribution, degradation, long-term retention, and clearance. Although promising magnetothermal release and therapeutic effects have been reported, evidence remains dominated by in vitro studies, with limited in vivo validation. Current clinical experience concerns locally administered iron-oxide hyperthermia rather than complete thermoresponsive Fe_3_O_4_@mSiO_2_ drug-delivery systems. Translation will require standardized magnetothermal and release testing, reproducible scale-up, validated sterilization and endotoxin control, component-resolved pharmacokinetics, and integrated development of the nanocarrier and AMF applicator.

## 1. Introduction

Nanotechnology has emerged as a transformative approach for the development of advanced drug delivery systems, offering opportunities to improve therapeutic control through nanoscale engineering of size, shape, surface chemistry, composition, and architecture. In contrast to conventional drug administration, nanocarriers can be engineered to protect therapeutic molecules from degradation, improve solubility and stability, modulate pharmacokinetics, and regulate release profiles [1,2,3]. They enable the selective delivery of therapeutic agents to specific tissues, thereby enhancing treatment efficacy while minimizing adverse effects [4].

Among the different classes of nanomaterials explored for biomedical applications, stimuli-responsive nanocarriers have attracted particular attention because they can regulate cargo release in response to endogenous or exogenous triggers. Endogenous stimuli-responsive systems are commonly activated by internal biological cues, such as pH, redox gradients, enzyme activity, or metabolite concentration, while exogenous stimuli-responsive systems are triggered by externally applied stimuli, including light, ultrasound, temperature, and magnetic fields [5,6]. External triggers are especially attractive because they provide spatial and temporal control over therapeutic activation, allowing drug release to be initiated only when and where required [7]. However, responsiveness alone is not sufficient to ensure effective therapeutic performance. An ideal stimuli-responsive drug delivery system should be based on biocompatible or biodegradable materials, provide high drug-loading capacity, promote selective delivery to the target site, prevent premature cargo leakage before activation, and enable precise release only in response to the intended endogenous or exogenous stimulus [8].

Magnetic fields represent particularly advantageous external triggers for controlled drug delivery because they can penetrate biological tissues non-invasively and can be applied and withdrawn on demand [9]. When magnetic nanoparticles are exposed to an alternating magnetic field (AMF), they convert magnetic energy into heat through relaxation and hysteresis losses, generating localized temperature increases without direct contact with the target tissue [10]. This magnetically induced heating can be used not only as a remote stimulus to activate thermoresponsive nanocarriers with high temporal precision, but also for standalone magnetic hyperthermia therapy [5,9]. Magnetic hyperthermia uses magnetic nanoparticles to generate heat, often targeting the mild hyperthermia range of approximately 40–45 °C; however, the biological effect depends on the full time-temperature history, spatial temperature heterogeneity, and tissue context. Such heating can directly stress tumor cells and can increase their sensitivity to chemotherapy or radiotherapy while limiting exposure of surrounding tissues [11].

Superparamagnetic iron oxide nanoparticles (SPIONs), particularly those composed of magnetite (Fe_3_O_4_), have been extensively investigated for a wide range of biomedical applications. Their ability to lose remanent magnetization once the external magnetic field is removed is particularly attractive, as it minimizes the risk of particle aggregation and improves colloidal stability in physiological environments [12]. Clinical use of ferumoxytol provides an important translational precedent for selected iron-oxide formulations. In the United States, ferumoxytol is marketed as Feraheme^®^ for intravenous iron replacement therapy [13], and under the brand FeraBright™ as an MRI contrast agent for adults with known or suspected malignant neoplasms in the brain [14].

Despite their advantages, the direct conjugation of drugs to SPION surfaces often results in limited loading capacity, premature drug release, and reduced magnetic heating efficiency due to alterations in surface chemistry [9,15]. To address these limitations, core–shell architectures combining magnetic cores with mesoporous silica (mSiO_2_) shells have been developed [9]. Mesoporous silica is characterized by high specific surface area, tunable pore architecture, and abundant surface silanol groups that facilitate functionalization [3,5]. The integration of SPIONs as magnetic cores with mesoporous silica shells creates multifunctional platforms that combine magnetic heating capability with high drug-loading capacity. This multifunctional platform can be further engineered with thermoresponsive polymers, which act as temperature-controlled gatekeepers by modulating pore accessibility in response to thermal changes [5].

Thermoresponsive polymers, particularly those exhibiting lower critical solution temperature (LCST) behavior such as poly(N-isopropylacrylamide) (PNIPAM) and poly(N-vinylcaprolactam) (PNVCL), undergo reversible coil-to-globule transitions at specific temperatures. When grafted onto mesoporous silica surfaces, these polymers can block pores in their swollen state below the LCST and collapse above the LCST, opening pores and triggering drug release [16,17,18,19].

Unlike reviews that discuss magnetic hyperthermia, mesoporous silica drug carriers, or thermoresponsive polymers as separate topics, this article integrates these elements around the central engineering problem of coupling efficient AMF heating to low-leakage, temperature-gated release in Fe_3_O_4_@mSiO_2_ architectures. The review therefore focuses on cross-component trade-offs: core immobilization and silica/polymer functionalization can alter heating; shell and pore designs affect loading, but also hydrodynamic size and magnetic dilution; and a polymer transition measured in the free state may not predict gating in physiological media. These design relationships are connected to integrated physicochemical validation, biological safety, manufacturability, component-resolved pharmacokinetics and degradation, and regulatory translation. The objective is to define experimentally testable criteria that distinguish a multifunctional proof-of-concept from a clinically credible magnetothermal drug-delivery system.

### Literature Search Strategy

This narrative review was informed by structured searches of Scopus, Web of Science, PubMed, and Google Scholar from database inception to 31 July 2026. Search terms combined “magnetic hyperthermia”, “AMF”, “SAR”, “Fe_3_O_4_@mSiO_2_”, “magnetic mesoporous silica”, “thermoresponsive polymer”, “PNIPAM”, “PNVCL”, “LCST”, “drug loading”, “controlled release”, “biodistribution”, “degradation”, and “clinical translation”, as appropriate. Priority was given to original studies reporting complete Fe_3_O_4_@mSiO_2_ or closely related magnetic mesoporous silica systems, while component-level evidence was included when direct data on the complete platform were unavailable. Clinical and regulatory sources were used to contextualize translational status, and reference lists of relevant articles were screened for additional studies. Because this article is a narrative rather than a systematic review, no formal meta-analysis or risk-of-bias assessment was performed.

## 2. Fundamentals of Magnetic Hyperthermia

### 2.1. Heat Generation Mechanisms in Superparamagnetic Nanoparticles

**Figure 1:**
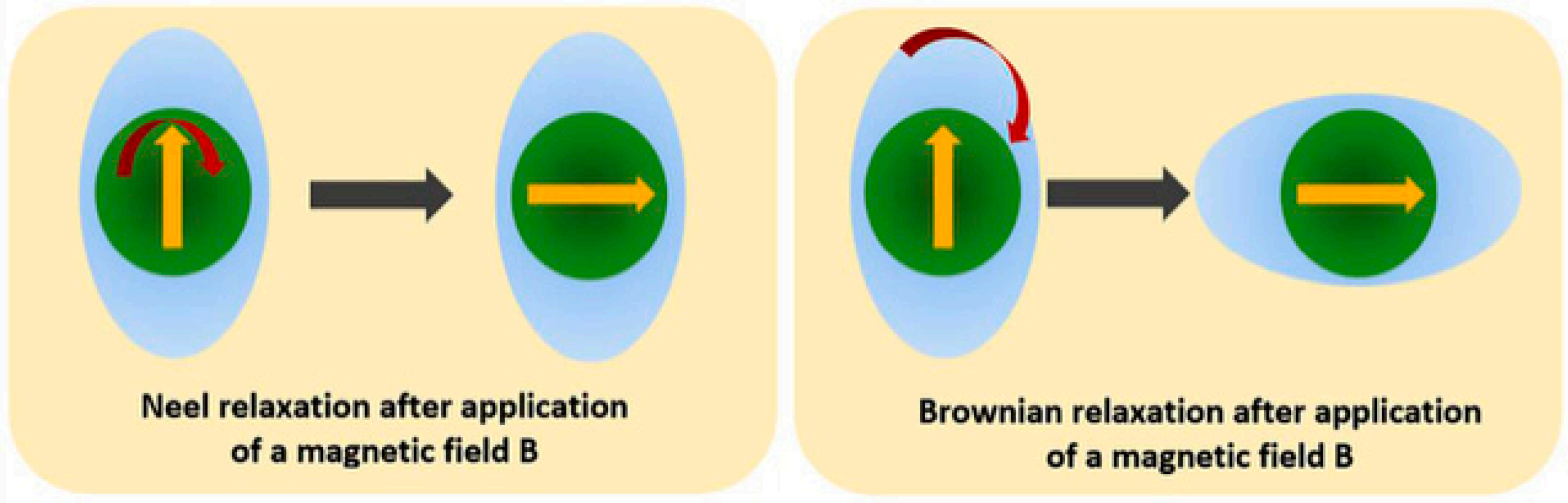
Schematic representation of heat-generation mechanisms in superparamagnetic nanoparticles under an alternating magnetic field. Reproduced from [22] under the terms of the Creative Commons license of the original publication.

When superparamagnetic iron oxide nanoparticles are exposed to an AMF, they generate heat through two primary mechanisms: Néel relaxation and Brownian relaxation. Néel relaxation involves the internal rotation of the magnetic moment within a fixed particle, while Brownian relaxation involves the physical rotation of the entire particle within the surrounding medium (Figure 1). Although these mechanisms are often discussed separately, they operate as competing parallel pathways, and their relative contributions depend on magnetic-core volume, effective magnetic anisotropy, hydrodynamic size, medium viscosity, temperature, interparticle interactions, and AMF frequency and amplitude [20,21].

For small magnetic cores (typically <10 nm for magnetite [22]), Néel relaxation tends to dominate, with the relaxation time $\tau_{N}$ given by:

(1) $$\tau_{N}=\tau_{0}e^{\frac{KV}{k_{B}T}}$$

where $\tau_{0}$ is the attempt time, K is the magnetic anisotropy constant, V is the magnetic core volume, $k_{B}$ is Boltzmann’s constant, and T is temperature. For larger particles, Brownian relaxation becomes significant, with relaxation time $\tau_{B}$:

(2) $$\tau_{B}=\frac{3\eta V_{H}}{k_{B}T}$$

where η is the medium viscosity and $V_{H}$ is the hydrodynamic volume [20,21].

Experimental evidence demonstrates that the balance between Néel and Brownian relaxation depends strongly on particle size and the surrounding environment. Vajtai et al. [23] investigated aqueous magnetite ferrofluids containing nanoparticles with diameters ranging from 6 to 13.5 nm. The 6 nm particles exhibited predominantly Néel-type behavior, whereas the 13.5 nm particles showed clear signatures of Brownian relaxation, including a low-frequency Debye-type peak in AC susceptibility and magnetic and thermal hysteresis near the freezing point of water. This Brownian-related susceptibility peak disappeared upon freezing, when whole-particle rotation was prevented, directly confirming the mechanical origin of this contribution. These findings indicate that Brownian relaxation becomes increasingly relevant with increasing particle size but contributes only when sufficient rotational mobility is retained.

The effects of viscosity and immobilization were further quantified by Avolio et al. [24] using magnetite nanoparticles with core diameters of approximately 10, 14, and 18 nm dispersed in either water or agarose gels. Immobilization had little effect on the SAR of the 10 nm particles, consistent with predominantly Néel-mediated heating. In contrast, the 14 and 18 nm particles exhibited substantial reductions in SAR after incorporation into the gel. For the 18 nm sample, SAR decreased from approximately 67 W g^−1^ in the aqueous suspension to 25 W g^−1^ in agarose gel at 110 kHz and 16.2 kA m^−1^. Measurements performed in low-viscosity dispersions may therefore overestimate the heating efficiency of nanoparticles that rely substantially on Brownian rotation under tissue-like confinement.

Biological interactions impose comparable constraints on particle rotation. Association with the cell membrane and subsequent internalization generally restrict whole-particle motion. Di Corato et al. [25] reported a rapid and systematic decrease in heating efficiency across several magnetic nanoparticle designs after their interaction with tumor cells, attributing this reduction to the complete suppression of Brownian relaxation under the investigated cellular conditions. Nevertheless, the degree of Brownian restriction is not uniform across all intracellular environments. Wang et al. [26] showed that identical amounts of Fe_3_O_4_ nanoparticles internalized by breast cancer cells with a softer and less organized cytoskeletal microenvironment exhibited a greater Brownian contribution and higher magnetothermal conversion than those internalized by mechanically stiffer normal mammary epithelial cells. These findings show that cell association and internalization generally attenuate Brownian relaxation by restricting whole-particle rotation, although the degree of restriction varies with nanoparticle design and the mechanical properties of the intracellular microenvironment.

Overall, particle size alone is insufficient to predict the dominant relaxation pathway; viscosity, immobilization, and the mechanical properties of the local environment must also be considered. Consequently, SAR values obtained in aqueous dispersions may not accurately predict heating under physiological conditions, an important limitation when interpreting the SAR and ILP metrics discussed below.

### 2.2. Specific Absorption Rate and Intrinsic Loss Power

The heating efficiency of magnetic nanoparticles is quantified by the specific absorption rate (*SAR*), defined as the power dissipated per unit mass of magnetic material:

(3) $$SAR=\;\frac{C}{m}\times \frac{∆T}{∆t}$$

where C is the heat capacity of the sample, m is the mass of magnetic material, and $\frac{∆T}{∆t}$ is the initial rate of temperature increase. *SAR* is typically reported in units of W/g and depends on the AMF amplitude (*H*), frequency (*f*), particle size, shape, composition, and dispersion state [21,27].

An alternative metric, the intrinsic loss power (*ILP*), normalizes *SAR* by the field amplitude and frequency:

(4) $$ILP=\frac{SAR}{H^{2}\times f}$$

This parameter removes the approximate linear dependence of *SAR* on frequency and its quadratic dependence on field amplitude. However, because the imaginary component of magnetic susceptibility is itself field- and frequency-dependent, *ILP* can only be considered approximately constant under relatively low-field and low-frequency conditions. It is therefore useful for comparing materials under AMF regimes relevant to clinical magnetic hyperthermia [27,28].

### 2.3. AMF Parameters and Safety Considerations

Safety considerations constrain the choice of AMF parameters. Increasing AMF amplitude or frequency can improve heating efficiency but may also induce nonspecific tissue heating through eddy currents. For this reason, safety-oriented criteria based on the product (H × f) are often used to assess whether AMF conditions are suitable for biomedical applications. The Atkinson–Brezovich limit (H × f ≤ 4.85 × 10^8^ A m^−1^ s^−1^ [29]) and the less restrictive Hergt–Dutz criterion (H × f ≤ 5 × 10^9^ A m^−1^ s^−1^ [30]) are commonly cited in magnetic hyperthermia studies. However, recent work has emphasized that these thresholds should not be interpreted as universal safety limits, since tolerability depends on multiple factors (e.g., exposed tissue volume, field geometry, anatomical location, exposure duration, and heat dissipation capacity), and further systematic studies are still needed [31,32].

Collectively, these considerations establish the basis for the material-design strategies discussed in the following section. An effective magnetothermal nanocarrier should retain sufficient magnetic energy dissipation while providing adequate silica porosity and a gatekeeper that remains closed at physiological temperature.

## 3. Design of Magnetothermally Responsive Fe_3_O_4_@mSiO_2_ Nanocarriers

### 3.1. Magnetic Core Functions and SAR Optimization

The magnetic core of Fe_3_O_4_@mSiO_2_ nanocarriers can perform several functions including: (i) heat generation under AMF exposure; (ii) contrast enhancement for magnetic resonance imaging; and (iii) magnetophoretic guidance [33,34]. The synthesis route and reaction conditions used to prepare the core are critical design variables because they control nanoparticle nucleation and growth and consequently influence the particle size and size distribution, morphology, phase composition, crystallinity, surface state, colloidal stability, and magnetic properties [35].

However, the synthesis route should not be regarded as an independent predictor of SAR. Rather, it determines a combination of structural and magnetic attributes, including magnetic volume, saturation magnetization, effective anisotropy, dipolar interactions, and colloidal organization, which collectively control magnetic losses under an AMF. The main advantages, limitations, and typical physicochemical outcomes of commonly used Fe_3_O_4_ synthesis strategies are compared in Table 1.

The selection of the most appropriate synthesis route depends on the intended critical quality attributes of the final nanocarrier. Thermal decomposition is often preferred when highly crystalline and monodisperse magnetic cores are required, although the resulting ligand-coated particles are generally hydrophobic and require phase transfer or surface modification before use in aqueous systems [43,44]. Conversely, co-precipitation is attractive because it is simple, inexpensive, and performed in aqueous media; however, simultaneous nucleation, growth, ripening, and coagulation can result in broad particle-size distributions [40]. Solvothermal synthesis provides flexibility for producing anisotropic structures such as Fe_3_O_4_ nanorods, whereas polyol routes are particularly suitable for preparing multicore assemblies such as magnetic nanoflowers [46,48]. Microemulsion methods favor ultrasmall particles with narrow size distributions but require surfactant-rich formulations and repeated purification steps [49]. Microwave irradiation can substantially shorten the reaction time of solvothermal and other nanoparticle synthesis routes. However, translation to continuous or larger-scale production requires careful optimization of reactor configuration, flow conditions, residence time, microwave power, and temperature distribution [51,52].

Among the properties determined by the synthesis route, particle size and morphology are particularly important because they influence magnetic-domain structure, magnetic anisotropy, relaxation dynamics, and ultimately heating efficiency, as discussed next. The magnetic behavior of iron oxide nanoparticles is strongly size-dependent and can be understood in terms of two main phenomena: the single-domain limit and the superparamagnetic limit. In bulk ferromagnetic or ferrimagnetic materials, magnetization is distributed across multiple magnetic domains separated by domain walls. The balance between magnetostatic energy and domain-wall energy governs the formation of these domains. As particle size decreases, the formation of domain walls becomes energetically unfavorable, and the particle adopts a single-domain configuration with uniform magnetization [53].

**Figure 2:**
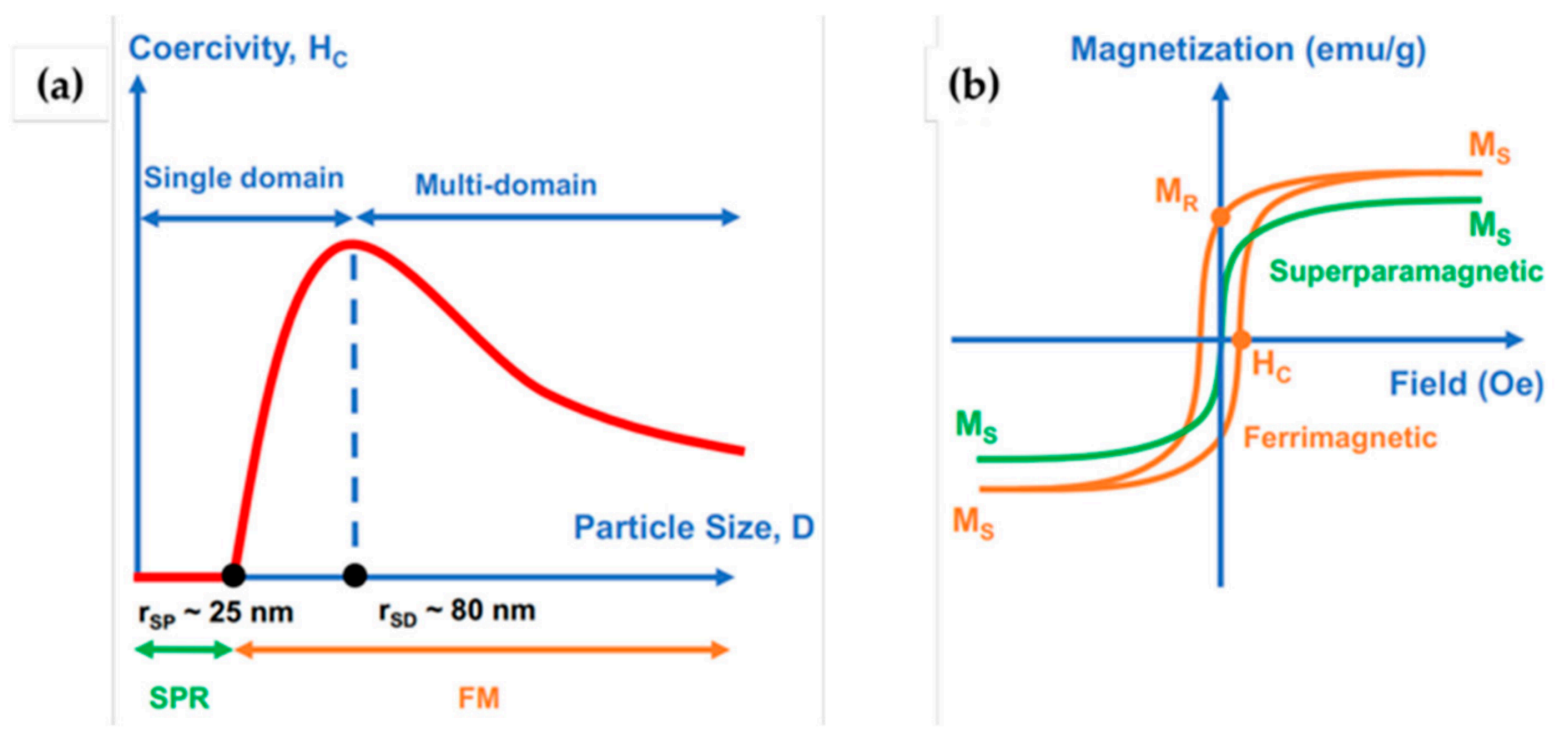
Size-dependent magnetic behavior of Fe_3_O_4_ nanoparticles. (**a**) Below the superparamagnetic threshold (r_SP_ ≈ 25 nm), thermal fluctuations result in negligible coercivity (H_c_). In the blocked single-domain regime, H_c_ increases with particle size, reaches a maximum near the transition to the multidomain regime (r_SD_ ≈ 80 nm), and subsequently decreases as domain-wall motion facilitates magnetization reversal. (**b**) Superparamagnetic nanoparticles exhibit a reversible magnetization response with negligible H_c_ and remanence (M_R_), whereas blocked ferrimagnetic nanoparticles display hysteresis with finite H_c_ and M_R_; M_S_ denotes the saturation magnetization. The indicated thresholds are approximate and depend on particle properties and measurement conditions. Reproduced from [54] under the terms of the Creative Commons license of the original publication.

Below a further critical size, thermal energy becomes sufficient to overcome the magnetic anisotropy energy barrier, allowing spontaneous reorientation of the magnetic moment. This phenomenon gives rise to superparamagnetism. Superparamagnetic iron oxide nanoparticles become magnetized under an external magnetic field but lose their net magnetization once the field is removed, showing negligible remanence, zero coercivity, and no hysteresis. This behavior is particularly advantageous for biomedical applications because it minimizes permanent magnetic interactions between particles and reduces the risk of irreversible aggregation [53]. These size-dependent magnetic regimes and their effects on coercivity are illustrated in Figure 2. As shown in Figure 2a, the coercivity of Fe_3_O_4_ nanoparticles varies nonlinearly with particle size. Nanoparticles below the superparamagnetic threshold (approximately 25 nm) exhibit negligible coercivity at room temperature when thermal fluctuations can readily reverse their magnetic moments. Above this threshold, the particles enter a magnetically blocked single-domain regime, in which coercivity increases with particle diameter. Coercivity reaches a maximum near the single-domain-to-multidomain transition, represented at approximately 80 nm. At larger sizes, the formation of multiple magnetic domains facilitates magnetization reversal through domain-wall motion, leading to a decrease in coercivity. Figure 2b further contrasts the reversible magnetization response of superparamagnetic nanoparticles with the hysteretic behavior of magnetically blocked ferrimagnetic particles, which exhibit finite coercivity and remanent magnetization [54].

In addition to size, core morphology is also a critical parameter for tuning the magnetic response and heating efficiency of Fe_3_O_4_-based nanocarriers. Compared with nearly spherical nanoparticles, anisotropic or faceted morphologies such as nanocubes, nanorods, octahedrons, plates, nano-octopods/deformed cubes, and multicore nanoflowers can introduce additional shape- and surface-anisotropy contributions to effective magnetic anisotropy. These contributions modify the energy barrier for magnetization reversal, the distribution of easy axes, and the dynamic hysteresis response under AMF exposure. Since the Néel relaxation time scales exponentially with the effective anisotropy energy barrier (Equation (1)), morphology-induced changes in effective anisotropy and magnetic volume can shift the optimal relaxation regime and alter the balance among Néel relaxation, Brownian rotation, and hysteresis-related losses [55,56,57].

Experimental studies have demonstrated that anisometric iron oxide nanoparticles can display shape-, orientation-, assembly-, and medium-dependent SAR values. Cubic Fe_3_O_4_ nanoparticles have shown superior heating performance compared with spherical counterparts, attributed in part to enhanced surface anisotropy [57,58]; magnetite nanorods with optimized aspect ratios can achieve markedly increased SAR [59]; and iron oxide nano-octopods or deformed cubes have been reported to increase SAR by approximately 70% relative to spherical nanoparticles of comparable size [60]. However, morphology alone is not sufficient to guarantee improved heating. Crystal structural integrity is essential, as cubic iron oxide nanoparticles with nearly identical size, morphology, and colloidal dispersion can exhibit markedly different magnetic hyperthermia responses when crystal lattice defects are present. Such defects may frustrate the orientation and dynamics of the magnetic moment within the cubic crystals, leading to weaker heating despite an otherwise favorable morphology [61]. Multicore nanoflowers further illustrate the role of morphology in enhancing magnetic heating through collective magnetic behavior arising from their multicore architecture and inter-core magnetic interactions. Their SAR has been shown to depend on the overall nanoflower diameter, morphology, number of constituting cores, structural defects, and AMF conditions, with multicore iron oxide nanoparticles generally exhibiting higher SAR than comparable single-core particles [58,62,63].

These effects are particularly relevant for magnetic mesoporous silica nanocarriers, where the silica framework can restrict whole-particle rotation and thereby suppress Brownian relaxation. In a magnetic mesoporous silica nanocomposite containing iron oxide nanoparticles, Zeleňáková et al. [64] showed that Brownian relaxation was suppressed by a “gripping effect” because rotation of the whole particle encapsulated within the mesoporous silica system was disabled; under these conditions, Néel relaxation became the dominant magnetic relaxation mechanism.

Importantly, shape-induced SAR enhancement is not universal. Therefore, SAR optimization in Fe_3_O_4_@mSiO_2_ nanocarriers should consider particle morphology together with core size, crystallinity, magnetic volume, interparticle spacing, surface coating, colloidal stability, and the degree of core immobilization within the silica framework.

Cation substitution can modify the saturation magnetization, coercivity, remanence, and magnetic anisotropy of spinel ferrites [65,66]. Co incorporation generally increases coercivity, whereas partial Zn substitution may enhance saturation magnetization [66]. However, these parameters alone do not predict magnetothermal performance. El-Boubbou et al. [67], who compared PVP-coated ferrites at 10 mg mL^−1^ under identical AMF conditions (332.8 kHz and 170 Oe), reported SAR values of 54.71 W g^−1^ for NiFe_2_O_4_ and 30.25 W g^−1^ for Fe_3_O_4_, whereas ZnFe_2_O_4_, MgFe_2_O_4_, and SnFe_2_O_4_ exhibited lower values of 23.50, 20.60, and 18.35 W g^−1^, respectively. Although this comparison controls the AMF conditions, differences in particle size and magnetic properties prevent the observed heating from being attributed solely to cation identity. Safety must also be considered. Oliveira et al. [68] detected metabolic alterations after exposure to CoFe_2_O_4_ despite limited conventional cytotoxicity, while Volatron et al. [69] demonstrated intracellular cobalt-ferrite degradation and transfer of released Co and Fe to ferritin. Substituted cores should therefore be evaluated for heating, dissolution, oxidative stress, metal-homeostasis disruption, retention, and clearance in the final mSiO_2_-coated formulation.

### 3.2. Mesoporous Silica Shell as Drug Reservoir

#### 3.2.1. Shell Thickness and Pore Architecture

The mesoporous silica shell in Fe_3_O_4_@mSiO_2_ nanocarriers provides a high-capacity drug reservoir with tunable pore architecture and can shield encapsulated cargo from premature degradation [3]. Mesoporous silica nanoparticles (MSNs) are characterized by high surface area (700–1300 m^2^/g), ordered or disordered pore structures with diameters typically in the 2–10 nm range, and abundant surface silanol groups (Si-OH) that facilitate functionalization [3,5].

Shell thickness is a key design parameter in Fe_3_O_4_@mSiO_2_ nanocarriers, as it directly influences drug-loading capacity, release kinetics, and magnetothermal performance. In general, the incorporation of a silica shell decreases the saturation magnetization when values are normalized to the total mass of the nanocarrier, mainly because a non-magnetic silica component is introduced around the Fe_3_O_4_ core [70]. However, the influence of shell thickness on the magnetic response is not necessarily linear and appears to be highly dependent on the specific core–shell architecture.

Vogt et al. [71] reported that increasing the silica shell thickness from 5.2 to 8.6 and 10.5 nm progressively decreased the saturation magnetization of the corresponding core–shell nanoparticles from 17.6 to 11.28 and 7.3 emu g^−1^ at 300 K, respectively. This trend was consistent with the increasing contribution of the non-magnetic silica fraction. In contrast, Taib et al. [72] observed a different behavior for Fe_3_O_4_@mSiO_2_ nanoparticles, where thinner mesoporous silica shells were associated with lower saturation magnetization values. In that study, decreasing the shell thickness from 73 to 63 and 45 nm was accompanied by a reduction in saturation magnetization from 83.6 to 56.9 and 43.4 emu g^−1^ at 300 K, respectively. The authors attributed this behavior to changes in interparticle magnetic interactions rather than to a simple magnetic dilution effect. Madrid et al. [73] further showed that coating Fe_3_O_4_ nanoparticles with a mesoporous silica shell reduced the saturation magnetization by approximately 25% compared with the uncoated magnetic core. However, within the shell thickness range evaluated in that study, further increases in shell thickness did not produce significant additional changes in saturation magnetization. These findings suggest that silica coating decreases the overall magnetic response by adding a non-magnetic component, but that moderate variations in shell thickness may not always strongly affect magnetization. Taken together, these findings indicate that a silica coating can decrease the overall magnetic response of Fe_3_O_4_-based nanocarriers by adding a non-magnetic component, but that moderate variations in shell thickness do not always produce proportional changes in magnetization. Therefore, shell thickness should be optimized not only to maximize drug-loading capacity and control release kinetics, but also to preserve sufficient magnetic responsiveness for AMF-triggered heating, magnetic guidance, or imaging applications.

#### 3.2.2. Safety of the Mesoporous Silica Interface

Although Fe_3_O_4_@mSiO_2_ nanocarriers are often described as biocompatible, the safety of the mesoporous silica shell is formulation- and exposure-dependent. Particle size, aggregation state, pore architecture, surface chemistry, residual template, degradation behavior, dose, and administration route jointly determine the biological response to the carrier [74,75,76,77,78].

The toxicity of mesoporous silica has mainly been associated with oxidative stress, inflammatory signaling, hemolysis, membrane damage, and genotoxic effects. Increased reactive oxygen species (ROS) production has been reported to disrupt cellular metabolism and contribute to lipid peroxidation, mitochondrial dysfunction, DNA damage, cell-cycle arrest, and apoptosis [74,75].

In mesoporous silica nanoparticles, surface silanol accessibility, pore structure, pore integrity, surface charge, and geometry have been shown to influence interactions with cell and erythrocyte membranes, affecting plasma membrane damage and hemolytic activity. Therefore, hemolysis and membrane-integrity assays are particularly relevant for evaluating the hemocompatibility of silica-based nanocarriers intended for intravenous administration, while oxidative stress assays should also be included based on the ROS-mediated toxicity mechanisms reported for silica nanoparticles [74,75,79,80].

Several physicochemical strategies have been investigated to mitigate MSN-associated toxicity, although their effects are formulation-dependent. Ji et al. [81] reported that hyaluronic acid-functionalized degradable dendritic MSNs exhibited lower hemolysis, nonspecific protein adsorption, in vitro cytotoxicity, and acute in vivo toxicity than unmodified or aminated particles. However, these improvements cannot be attributed exclusively to hyaluronic acid because functionalization also altered surface charge, colloidal stability, protein adsorption, and potentially cellular uptake.

Pore architecture has also been implicated in MSN biocompatibility, although its effects are difficult to distinguish from those of correlated physicochemical properties. Grunberger et al. [76] compared similarly sized (~100 nm) nonporous Stöber particles (SNP100), conventional MSNs (Meso100), and hollow MSNs (HMSNP100). At 500 µg/mL, SNP100 and HMSNP100 were hemolytic, whereas Meso100 remained non-hemolytic. Only HMSNP100 induced approximately 25% platelet aggregation and prolonged APTT, while SNP100 weakly activated complement. Nevertheless, Meso100 exhibited the greatest myelosuppressive potential and induced leukocyte procoagulant activity at the same concentration. Thus, Meso100 showed better direct hemocompatibility but a less favorable response in some immune-cell assays, demonstrating that conclusions based on a single toxicity endpoint may be misleading. These differences also cannot be attributed exclusively to pore size because the particles varied in accessible surface area, pore volume, hydrophobicity, and internal architecture. Furthermore, the adverse effects occurred mainly at 500 µg/mL; the absence of detectable immunotoxicity at lower concentrations does not establish chronic or repeated-dose safety.

Pérez-Moreno et al. [82] compared MSNs with pore diameters ranging from 3.19 to 11.3 nm. The smallest-pore particles showed greater dendritic-cell uptake and were the only unloaded formulation to reduce cell viability significantly. However, pore diameter varied together with surface area, pore volume, surface charge, and drug-loading and release behavior. Consequently, the results do not demonstrate that increasing pore size is intrinsically protective or define a universally safe pore diameter.

Residual synthesis reagents represent an additional safety concern. Cetyltrimethylammonium bromide (CTAB) is frequently used as a structure-directing agent during mesoporous silica synthesis. Although CTAB enables pore formation, incomplete surfactant removal may increase cytotoxicity. Therefore, surfactant-removal procedures should be optimized, and CTAB removal should be confirmed using appropriate analytical techniques before biological testing [77,83]. In Fe_3_O_4_@mSiO_2_ systems, incomplete template removal may confound toxicity results, making it difficult to distinguish intrinsic silica-related effects from surfactant-induced toxicity.

Overall, surface coatings designed to improve biocompatibility, optimization of pore architecture, and rigorous removal of residual synthesis reagents may reduce MSN-associated toxicity, but their effectiveness must be evaluated in the complete Fe_3_O_4_@mSiO_2_ nanocarrier. The biological response may be modified by the magnetic core, silica-shell thickness, polymer gatekeeper, loaded drug, and any residual contaminants through their effects on the accessible silica surface, aggregation, protein-corona formation, degradation, and cellular uptake.

#### 3.2.3. Drug-Loading Mechanisms

Drug loading can be classified according to the dominant retention mechanism and the processing route. Retention modes include (i) non-covalent adsorption and nanoconfinement within the mesopores, (ii) covalent conjugation to the silica surface or pore walls, and (iii) encapsulation within an additional polymeric or lipid phase; hybrid systems may combine these modes [2]. Processing routes include solvent-based methods, such as adsorption, incipient-wetness impregnation, solvent evaporation, vacuum-assisted impregnation, co-spray drying, and supercritical-fluid impregnation, as well as solvent-free methods, including melt loading, microwave-assisted loading, and co-milling. Drug incorporation during MSN synthesis has also been reported [3,84].

In physically loaded systems, hydrogen bonding, electrostatic attraction, Van der Waals forces, hydrophobic interactions, and nanoconfinement govern drug retention. Loading and release therefore depend on the drug/carrier ratio, solvent, pH, pore volume and accessibility, surface area, and drug–silica affinity. Physical loading is comparatively simple, broadly applicable, and does not require drug derivatization. However, it is sensitive to processing conditions and may result in external drug crystallization, premature leakage, burst or incomplete release, and residual-solvent contamination. Polymer coatings and pore gatekeepers can reduce leakage but add formulation complexity and occlude pore entrances until activation [2,3,84].

Ostrowska et al. [85] illustrated adsorption-based loading using polydopamine-coated MSNs containing doxorubicin or sorafenib. Doxorubicin was loaded in phosphate-buffered saline, whereas sorafenib required dimethylformamide because of its limited aqueous solubility, yielding loading capacities above 66 and approximately 65.2 wt%, respectively. Because adsorption-based values depend strongly on the initial drug concentration, comparisons between formulations should consider the drug/carrier ratio, loading-capacity definition, and removal of unbound drug [3,84,85].

**Figure 3:**
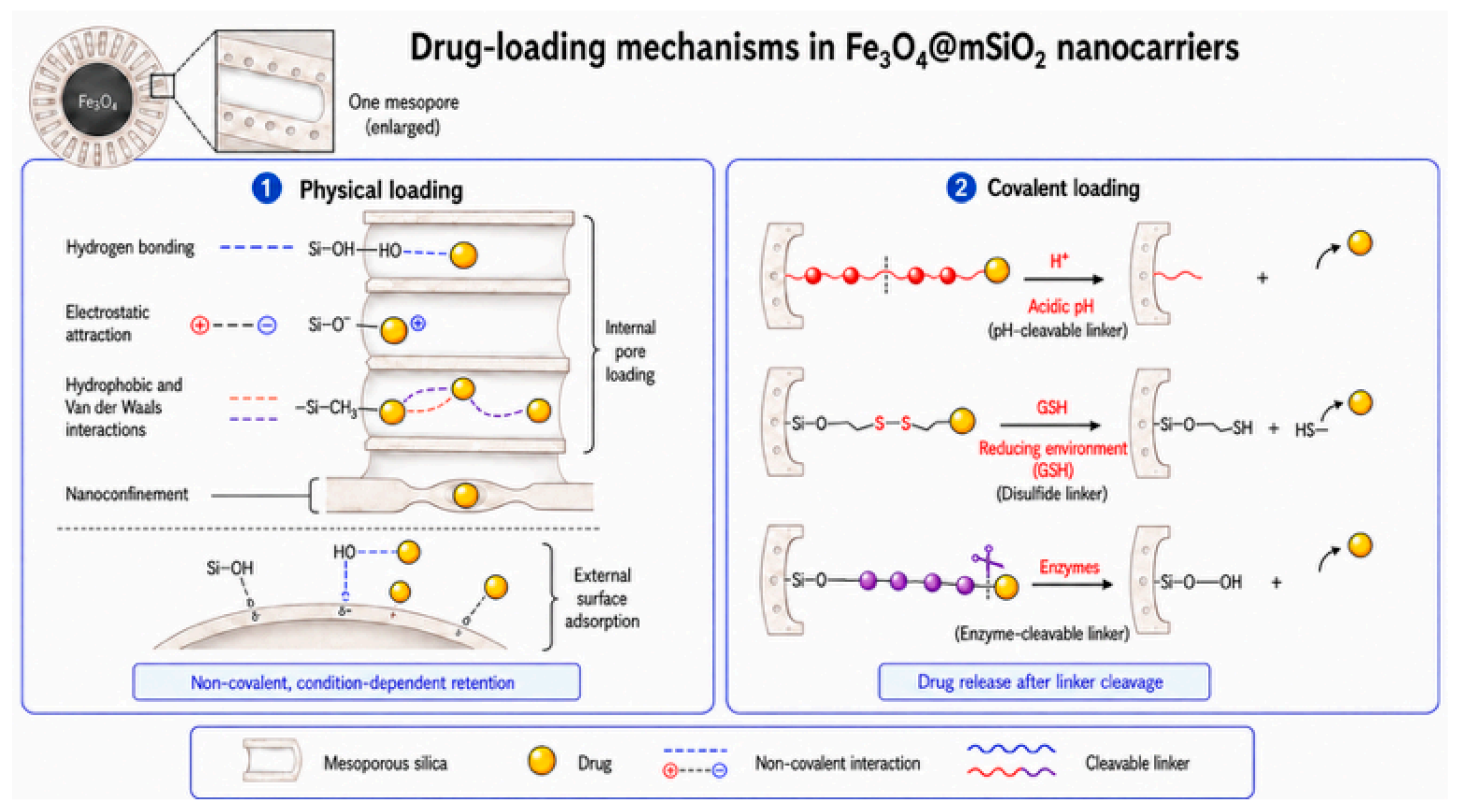
Schematic representation of physical and covalent drug-loading mechanisms in Fe_3_O_4_@mSiO_2_ nanocarriers. The illustrated interactions and cleavable linkers are representative and depend on drug and surface chemistry.

Covalent grafting can reduce premature leakage and enable controlled release through stimulus-cleavable linkers. Hydrazone, imine, or acetal linkages can promote release at acidic pH, disulfide linkers respond to intracellular reducing conditions, and peptide linkers can enable enzyme-mediated release. Compared with physical loading, covalent conjugation provides greater control over drug retention, stoichiometry, and release but requires suitable reactive groups or drug-derived prodrugs, multistep processing, and the optimization of conjugation and cleavage efficiencies. Incomplete cleavage may limit recovery of the active drug, while extensive functionalization may reduce pore accessibility or consume groups required for polymer, gatekeeper, or targeting-ligand attachment. Hybrid strategies combine the versatility of physical loading with the control provided by covalent attachment but increase formulation complexity, possible pore obstruction, scale-up difficulty, and quality-control requirements [2,86]. The principal interactions governing physical drug loading and representative pH-, redox-, and enzyme-cleavable linkers used for covalent loading are summarized in Figure 3.

Yan et al. [87] demonstrated a hybrid strategy in which doxorubicin was physically loaded into MSN pores, whereas paclitaxel was covalently attached through a disulfide-containing linker, enabling redox-triggered release. Tarannum et al. [86] subsequently developed an MSN platform in which cisplatin and gemcitabine prodrugs were selectively conjugated to the inner and outer surfaces, respectively, through reduction-sensitive linkages. PEGylation and TAB004 functionalization enabled tMUC1-targeted, spatiotemporally controlled, and ratiometric co-delivery in pancreatic ductal adenocarcinoma (PDAC) models. Further optimization showed that among the dual-drug formulations, the formulation containing 10 wt% gemcitabine and 9 wt% cisplatin produced the strongest cytotoxicity despite its lower total drug content. More heavily loaded formulations exhibited poorer internalization, associated with less favorable colloidal properties, demonstrating that maximum loading does not necessarily maximize in vitro activity [88].

For Fe_3_O_4_@mSiO_2_ magnetothermal nanocarriers, loading performance should be evaluated in the complete formulation because the magnetic core reduces the silica mass fraction, while polymer grafting or covalent conjugation may restrict pore accessibility or consume surface-reactive groups. Loading capacity should therefore be reported relative to the total nanocarrier mass, and where possible, the silica mass, together with encapsulation efficiency, initial drug/carrier ratio, removal of unbound drug, and storage stability.

### 3.3. Synthesis Approaches and Architecture Control of mSiO_2_ Shell

The synthesis of Fe_3_O_4_@mSiO_2_ core–shell nanoparticles typically involves two main steps: (i) synthesis of the magnetic cores, and (ii) coating with mesoporous silica. Several synthetic routes for (ii) have been developed, each with distinct advantages and limitations. The Stöber method, based on base-catalyzed hydrolysis and condensation of tetraethyl orthosilicate (TEOS) in ethanol/water mixtures, is the most widely used approach for coating magnetic cores with silica shells [5,89,90]. The physicochemical properties can be controlled by adjusting TEOS concentration, reaction time, temperature, pH, solvent type and amount, and ammonia concentration [90]. To introduce mesoporosity, surfactant templates (e.g., CTAB) are added during the sol–gel process, followed by template removal via calcination or solvent extraction [5,89,90]. Variations of this method include the use of pore-swelling agents or co-templates to tune pore size [91], as well as the incorporation of functional organosilanes during synthesis to introduce reactive groups such as amine, thiol, or carboxyl moieties [92,93].

Other approaches, such as reverse microemulsion, have also been explored to improve control over particle size and shell uniformity. For instance, Asgari et al. developed a modified reverse microemulsion route to produce monodisperse Fe_3_O_4_@mSiO_2_ core–shell nanoparticles with tunable silica shell thickness, mesoporosity, superparamagnetic behavior, and potential application as 5-fluorouracil carriers [70].

### 3.4. Thermoresponsive Polymer Gatekeepers

Premature drug leakage remains one of the main limitations of mesoporous silica-based delivery systems. Although mesoporous silica provides high surface area and pore volume for cargo encapsulation, its open pore network can also allow uncontrolled diffusion under physiological conditions unless the pore entrances are properly capped [4,5,8]. Gatekeeper systems are therefore used to block the mesopores during circulation and to enable release only after exposure to a specific stimulus, such as pH, redox conditions, enzymes, light, magnetic fields, or temperature [8].

**Figure 4:**
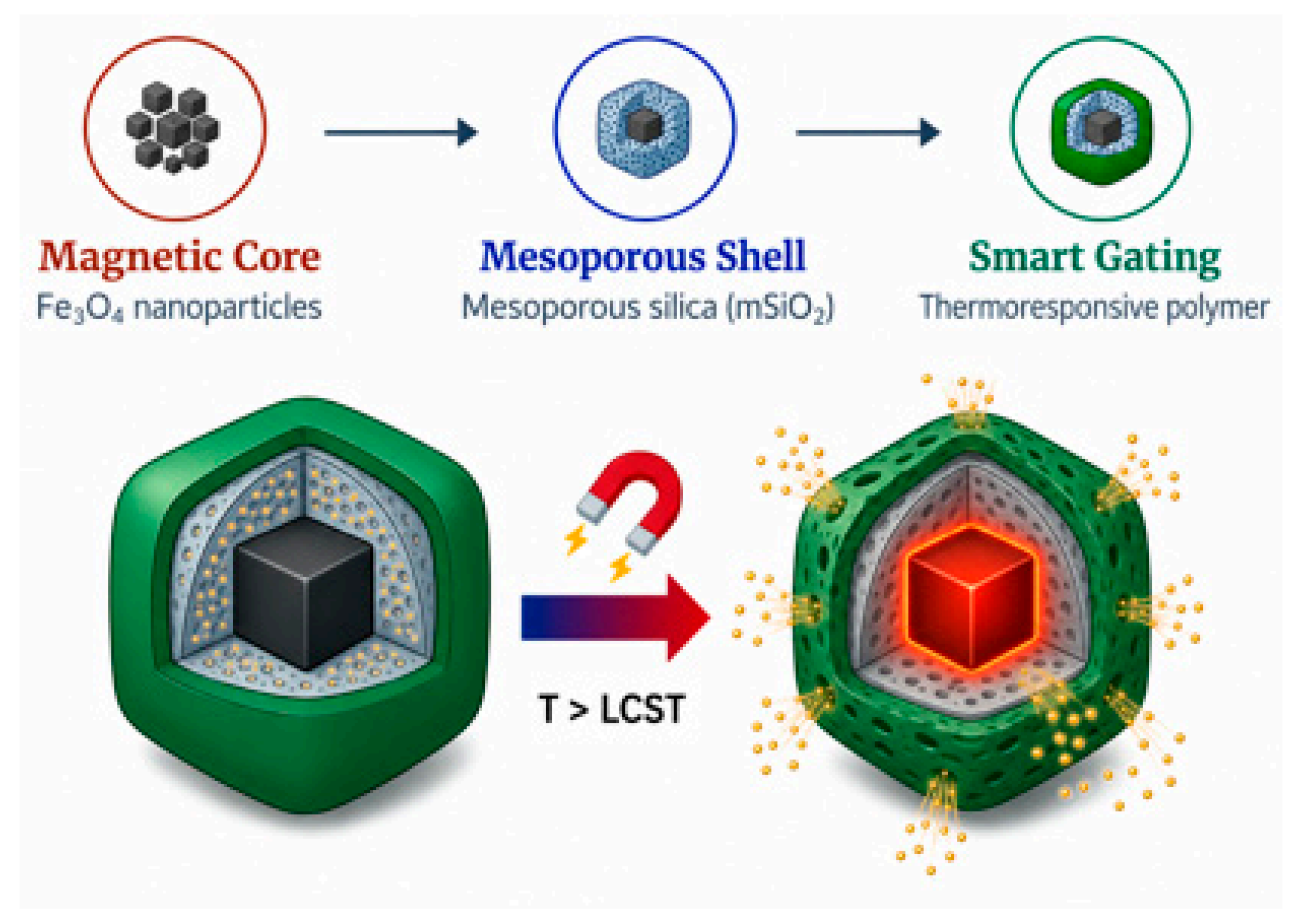
Schematic illustration of AMF-triggered drug release. Application of an AMF induces localized heating in the Fe_3_O_4_ core. Once the temperature exceeds the polymer’s LCST, the polymer chains collapse, opening the mesoporous silica pores and enabling controlled drug release.

In magnetothermally responsive Fe_3_O_4_@mSiO_2_ nanocarriers, thermoresponsive polymers are especially useful because they can convert AMF-induced heating into controlled pore opening. Below their LCST, these polymers are hydrated and swollen, limiting cargo diffusion through the pore entrances. Above the LCST, they collapse, increasing pore accessibility and promoting drug release (Figure 4) [5,94]. For magnetothermal drug delivery, the gatekeeper should therefore combine low leakage at 37 °C, activation within the mild hyperthermia range, preferably 40–45 °C, reversible swelling/collapse behavior, stability in physiological media, and minimal interference with magnetic heating.

Thermoresponsive polymers can be grafted onto mesoporous silica through “grafting-to” or “grafting-from” strategies. In the grafting-to approach, pre-formed polymer chains are attached to the particle surface through reactive end groups, allowing better control over polymer molecular weight but often limited by steric hindrance. In the grafting-from approach, polymer chains are grown from surface-immobilized initiators or chain-transfer agents, which can improve surface coverage but may be affected by termination reactions and the limited accessibility of reactive sites [95]. In both cases, polymer molecular weight, grafting density, and chain length must be optimized to balance pore blocking and triggered release.

PNIPAM is the most widely studied thermoresponsive gatekeeper because of its well-characterized coil-to-globule transition and an LCST near 32 °C in dilute aqueous solution. This nominal transition is close to physiological temperature and below the usual mild-hyperthermia window, which can increase the risk of premature leakage [5,16].

Therefore, PNIPAM is often copolymerized with hydrophilic monomers to increase the LCST and shift release toward 40–45 °C. For example, PNIPAm-PAAm-modified mesoporous silica nanoparticles showed limited release under physiological conditions and enhanced release at elevated temperature and acidic pH [16]. Other PNIPAM-based copolymers, including poly(N-isopropylacrylamide-co-methacrylic acid) (P(NIPAM-co-MAA)) [96], poly(N-isopropylacrylamide-co-acrylic acid) (P(NIPAM-co-AA)) [97], and poly(N-isopropylacrylamide-co-3-methacryloxypropyltrimethoxysilane) (P(NIPAM-co-MPS)) [98], and chitosan-g-NIPAAm [99] have also been explored to tune LCST, introduce additional responsiveness, or improve biocompatibility.

Poly(N-vinylcaprolactam) (PNVCL) is an attractive alternative to PNIPAM because it can exhibit LCST-type behavior close to physiological or hyperthermic temperatures and offers broader tunability. Unlike PNIPAM, whose LCST is relatively constant around 32 °C, PNVCL commonly shows LCST values between 25 and 50 °C, depending on molecular weight, dispersity, concentration, and medium composition, including salts and proteins [100].

Besides PNIPAM and PNVCL, other known LCST thermoresponsive polymers include poly(glycidyl methyl ether-co-glycidyl ethyl ether) (P(GME-co-GEE)), poly(oligo(ethylene glycol) methyl ether methacrylate) (POEGMA), poly(ethylene glycol) (PEG), poly(propylene glycol) (PPG), and elastin-like polypeptides (ELPs) [101].

### 3.5. Integrated Structure–Property Relationships

The functional performance of Fe_3_O_4_@mSiO_2_ nanocarriers cannot be inferred from the properties of their individual components alone because successive assembly and functionalization steps modify the composition, architecture, colloidal organization, and biological interface of the complete formulation. Magnetic-core size, crystal integrity, magnetic-domain structure, and core organization jointly determine intrinsic magnetothermal performance [61,62]. However, aggregation, immobilization, and intracellular confinement can modify magnetic relaxation and reduce heating efficiency relative to freely dispersed nanoparticles [25]. Silica deposition increases the overall particle dimensions and introduces a nonmagnetic fraction that affects magnetization when values are normalized to the total nanocarrier mass [71]. Moreover, changes in silica-shell thickness and pore architecture can alter porosity, colloidal organization, and magnetic heating, although these effects do not necessarily follow a monotonic dependence on shell thickness [33].

Subsequent polymer functionalization can modify hydrodynamic size, accessible surface area, pore accessibility, and temperature-dependent transport through the mesopores [98,102]. Thermoresponsive polymer coatings can suppress passive leakage below their transition temperature and enhance release following polymer collapse or rearrangement above the LCST [94,98,102]. The amount and architecture of the polymer shell depend on molecular weight, grafting strategy, steric hindrance, and termination reactions during surface polymerization [95]. Polymer composition, molecular weight, concentration, and the surrounding medium can also shift or broaden the apparent thermal transition [100,103]. Surface functionalization can modify protein adsorption, hemocompatibility, cytotoxicity, and cellular internalization [81], as well as circulation and organ distribution [78]. Surface coatings can additionally alter the dissolution and degradation behavior of the silica component [104].

These coupled relationships complicate the interpretation of individual performance metrics. SAR normalized to iron or magnetic-material mass characterizes the heating efficiency of the magnetic component. In contrast, normalization to the complete nanocarrier mass includes the nonmagnetic silica, polymer, and cargo fractions in the denominator and is therefore not directly equivalent to iron- or magnetic-material-normalized SAR. Meaningful interpretation also requires reporting the AMF amplitude and frequency, particle concentration, calorimetric configuration, normalization basis, and data-analysis method [71,105,106]. The dispersion medium, aggregation state, and immobilization or intracellular confinement must also be considered because they can alter the available magnetic-relaxation mechanisms and heating efficiency [25].

A high nominal surface area or pore volume does not necessarily ensure high drug loading, which also depends on accessible porosity, drug–silica affinity, drug concentration and molecular state, solvent properties, and the loading, washing, and purification procedures [107]. Polymer functionalization can modify pore accessibility through the grafting strategy, polymer coverage, and organization of the surface layer and may provide an external gate that restricts diffusion through the pore openings [95,98,102]. Release kinetics depend on drug localization within the silica, drug–surface interactions, and pore-mediated diffusion [84,107], as well as on the response of the polymer gate and the temperature and pH of the release medium [94,102].

Therapeutic efficacy consequently cannot be inferred from a single SAR, loading capacity, or cumulative-release value. The delivered magnetic-material and drug doses, together with the spatial distribution of the carrier, determine the amount of heating and therapeutic cargo available within the tumor [108]. Cellular association and intracellular confinement can modify magnetothermal performance [25], while intracellular localization may enable local magnetic heating to be combined with triggered drug release [109]. The applied AMF conditions, achieved temperature, exposure duration, and resulting release profile collectively define the implemented chemohyperthermia protocol [108,109]. Therapeutic outcomes also depend on nanocarrier design, treatment modality, and the biological model used for evaluation [110].

Consequently, optimization should combine component-specific characterization with validation of the fully assembled nanocarrier under biologically relevant conditions. Performance metrics should be interpreted collectively because modifications that improve one function may compromise another, and properties measured for isolated components may not be retained after their integration into the complete formulation.

## 4. Physicochemical Characterization and Validation of Fe_3_O_4_@mSiO_2_ Magnetothermal Nanocarriers

The design of Fe_3_O_4_@mSiO_2_ magnetothermal nanocarriers requires not only the successful synthesis of a magnetic core, a mesoporous silica reservoir, and a thermoresponsive gatekeeper, but also the systematic validation of each functional component. Because these platforms are intrinsically multifunctional, no single characterization technique is sufficient to confirm their performance. Instead, structural, colloidal, textural, magnetic, thermoresponsive, and drug-release properties should be systematically evaluated in an integrated manner before proceeding to biological characterization. This is particularly important for AMF-triggered drug delivery systems, where small changes in shell thickness, aggregation state, polymer grafting density, or biological medium composition can strongly affect heating efficiency, pore accessibility, and release kinetics.

### 4.1. Morphology, Size, and Colloidal Stability

Electron microscopy is commonly used to assess the morphology and architecture of mesoporous silica-based nanocarriers. Transmission electron microscopy (TEM) is particularly useful for visualizing internal structural features, allowing for estimation of the magnetic core diameter, silica shell thickness, and in some cases, mesopore organization. Scanning electron microscopy (SEM) provides complementary information on particle morphology and surface features, whereas high-resolution transmission electron microscopy (HRTEM) can offer higher-resolution insight into crystalline domains and fine structural details, including pore arrangement and possible changes after surface modification or cargo loading [107]. However, because electron microscopy is performed on dried samples, dynamic light scattering (DLS) and zeta potential measurements are also needed to evaluate hydrodynamic size, aggregation, and colloidal stability in suspension. These analyses should be performed not only in water, but also in PBS, cell culture medium, and serum-containing media, since ionic strength influences colloidal stability, while serum proteins rapidly adsorb onto nanoparticle surfaces, forming a protein corona that alters their biological identity and cellular interactions [111].

### 4.2. Mesoporosity and Surface Chemistry

Nitrogen adsorption–desorption analysis is commonly used to determine Brunauer–Emmett–Teller (BET) surface area, pore volume, and pore size distribution, frequently using Barrett–Joyner–Halenda (BJH) or related models for pore size analysis. These parameters are directly linked to drug-loading capacity and release behavior. However, they should ideally be measured not only for the empty Fe_3_O_4_@mSiO_2_ carrier, but also after polymer grafting, and where possible, after drug loading, since pore blocking or partial collapse of accessible porosity may occur during functionalization [107].

Surface chemistry should be studied using complementary techniques. Fourier transform infrared spectroscopy (FTIR) can identify characteristic Si–O–Si, Si–OH and organic functional groups, while thermogravimetric analysis (TGA) can estimate the amount of organic material associated with the surface, including grafted polymers or molecular gatekeepers [112]. In CTAB-templated mesoporous silica systems, confirmation of surfactant removal is particularly important, since residual CTAB can strongly influence cytotoxicity and confound biological interpretation. Therefore, template removal should be verified before drug-loading and cell-based assays [77,83].

### 4.3. Magnetic and Magnetothermal Characterization

Magnetic properties are commonly evaluated by vibrating sample magnetometry (VSM) or superconducting quantum interference device (SQUID) magnetometry, which provide saturation magnetization, coercivity, and remanence. Superparamagnetic-like behavior, with low coercivity and remanence, is generally desirable for biomedical applications [113].

X-ray diffraction (XRD) can be used to confirm the crystalline iron oxide phase, although distinguishing magnetite from maghemite may require additional techniques such as Mössbauer spectroscopy, Raman spectroscopy, or X-ray photoelectron spectroscopy (XPS) [114].

Magnetothermal performance is usually assessed by AMF calorimetry and reported as SAR. Because the measured value is strongly affected by field amplitude and frequency, nanoparticle and iron concentration, aggregation, medium viscosity, sample geometry, heat losses, and the analysis method, cross-study comparisons require complete reporting. At minimum, studies should state H, f, H × f, sample volume, iron and total nanocarrier concentration, and the mass-normalization basis [21,27,28,105].

### 4.4. Thermoresponsive Gatekeeper and Drug Release Validation

For thermoresponsive Fe_3_O_4_@mSiO_2_ nanocarriers, the polymer gatekeeper must be characterized both chemically and functionally. The relevant transition temperature should be determined for the grafted nanocarrier, using temperature-dependent DLS or turbidimetry, UV–Vis spectroscopy, or differential scanning calorimetry (DSC).

In addition to LCST determination, the amount and architecture of the grafted polymer should be evaluated. TGA can estimate the total organic content, while FTIR and XPS can confirm successful functionalization [103].

Drug loading and release should be quantified using a validated method appropriate to the cargo, such as UV–Vis or fluorescence spectroscopy or high-performance liquid chromatography (HPLC). Passive leakage at 37 °C should be measured over a relevant time scale, and AMF-triggered release should be reported together with the achieved temperature and thermal history [107,115].

Table 2 provides an overview of the key physicochemical parameters and characterization techniques relevant to nanocarrier development.

## 5. Biological Safety, Biodistribution, Degradation, and Clearance

Following physicochemical validation, the biological safety of Fe_3_O_4_@mSiO_2_ magnetothermal nanocarriers cannot be inferred from short-term cell-viability assays alone. Because the magnetic core, mesoporous silica shell, polymer coating, targeting ligands, therapeutic cargo, and residual synthesis reagents may independently or synergistically influence biological responses, a formulation-specific assessment is required [116]. The final formulation should therefore be compared with relevant intermediate controls, such as the bare Fe_3_O_4_ core, Fe_3_O_4_@mSiO_2_, the coated but unloaded carrier, and the free therapeutic cargo. Dose, exposure duration, administration route, hydrodynamic size, aggregation state, and stability in biological media should also be reported.

Hemocompatibility studies have produced contrasting results. Achilli et al. [117] reported hemolytic, pro-thrombotic, and pro-inflammatory effects of silica-coated magnetite nanoparticles, whereas Shao et al. [118] observed no significant cytotoxicity or hemolysis among magnetic mesoporous silica nanoparticles with different shapes. More recently, Grunberger et al. [76] showed that silica size, porosity, and architecture can differentially affect hemolysis, complement activation, platelet aggregation, and plasma coagulation. Hemocompatibility must therefore be established for each final formulation using complementary assays, including hemolysis, platelet activation or aggregation, coagulation times, and complement activation [76,116].

Immunogenicity remains insufficiently investigated for complete Fe_3_O_4_@mSiO_2_ nanocarriers. Functionalized mesoporous silica nanoparticles have produced either limited immune-cell activation or adjuvant-like effects involving lymphocyte proliferation, cytokine secretion, antibody production, and long-term T-cell memory, depending on the formulation and experimental context [119,120]. Mesoporous silica should therefore not be assumed to be immunologically inert. Innate and adaptive responses should be examined using cytokine profiling, immune-cell phenotyping, complement assays, and, particularly in repeated-dose studies, anti-carrier antibody and T-cell-response measurements [116].

Oxidative stress is strongly influenced by core–shell stability and surface functionalization. In an in vitro model using iron oxide nanoparticles coated with a thin, dense silica layer rather than a mesoporous shell, surface passivation increased resistance to acidic degradation and reduced intracellular iron release, disruption of iron homeostasis, ROS production, mitochondrial dysfunction, cytotoxicity, and genotoxicity [121]. Thus, the presence of a silica shell does not itself guarantee protection; its chemical stability, porosity, and surface passivation are also critical. Oxidative stress assessment should complement viability assays with measurements of ROS and redox status, lipid peroxidation, mitochondrial and lysosomal integrity, DNA damage, and iron-homeostasis markers.

Direct in vivo studies confirm that Fe_3_O_4_@mSiO_2_ biodistribution is formulation-dependent. Rascol et al. [78] found no evident renal, hepatic, inflammatory, or histological toxicity four days after the intravenous administration of 40 mg kg^−1^, but the surface coating altered blood persistence and organ deposition. DMPC-coated particles accumulated more strongly in the liver and less in the lungs, whereas PEGylated particles remained detectable in blood for longer. However, the short follow-up precluded conclusions regarding chronic toxicity or complete clearance. Shao et al. [118] found predominant accumulation in the liver, spleen, and kidneys, with greater hepatic retention of spherical particles and greater splenic retention, cellular internalization, and tumor accumulation of long rods. Long-term studies indicate that apparently favorable acute results do not exclude delayed effects. In a silica-coated iron oxide model, Oziri et al. [122] observed dose-dependent hematological, renal, and hepatic alterations at the highest dose, while splenic iron remained elevated 28 days after administration. In a one-year study of silica nanoparticles, routine blood biomarkers were largely unchanged despite microscopic tissue alterations, particularly for larger non-porous particles; however, the mesoporous formulations did not show statistically significant chronic toxicity [123]. These findings demonstrate the need to combine blood biomarkers with organ histology and sufficiently long follow-up periods.

Direct evidence regarding the degradation of complete Fe_3_O_4_@mSiO_2_ nanocarriers remains limited. In PBS, the mesoporous silica shell dissolved from the inner region, forming a transient rattle-type structure, while the PEI coating delayed degradation [104]. In a non-magnetic core–shell silica model, the different radiotracer distributions observed for core- and shell-labelled nanoparticles reflected the rapid release of ^89^Zr from the degrading mesoporous shell compared with the greater short-term stability of the core-embedded label. These results demonstrate that radiolabel position must be considered when interpreting positron emission tomography (PET)-based biodistribution and can be exploited to monitor silica degradation [124]. Evidence from the individual components indicates that Fe_3_O_4_ degradation releases bioavailable iron that can be incorporated into endogenous transport, storage, and erythropoietic pathways [125]. Mesoporous silica, in contrast, hydrolyses into soluble silicic acid species. In one MSN formulation, degradation-derived silicic acid was detected in urine and feces, with elimination reported within four days after intravenous administration [126]. These results remain formulation-specific because degradation depends strongly on silica condensation, porosity, coating, and biological environment.

Accordingly, the safety assessment should integrate component-resolved biodistribution and clearance with histological and physicochemical analyses. Quantification of iron and silicon in blood, organs, urine, and feces should be complemented by Prussian blue staining, magnetic measurements, and microscopy. Where feasible, independent and stable core and shell labels should be employed to distinguish intact nanocarriers from separated components, degradation products, or detached labels [124]. Acute studies should be extended to repeated-dose designs and sufficiently long follow-up periods [122,123]. Overall, safety conclusions must remain formulation-, dose-, and route-specific and should encompass hemocompatibility, immunogenicity, oxidative stress, biodistribution, degradation, long-term retention, and clearance.

## 6. Antitumor Effects Using Combined Magnetic Hyperthermia and Chemotherapy

A major advantage of magnetothermally responsive Fe_3_O_4_@mSiO_2_ nanocarriers is their ability to combine magnetic hyperthermia therapy with controlled chemotherapy delivery, exploiting synergistic mechanisms to enhance therapeutic efficacy against cancer [127,128]. Mild hyperthermia, typically within the 40–45 °C range, can potentiate chemotherapy by improving tumor perfusion and facilitating drug accumulation, uptake, and release. In parallel, heat-induced cellular stress can intensify drug cytotoxicity by damaging proteins and other macromolecules, impairing DNA repair, promoting apoptosis, and attenuating multidrug-resistance mechanisms such as P-glycoprotein-mediated efflux [10,129]. Simultaneous or sequential delivery of chemotherapy during hyperthermia can produce synergistic cytotoxicity while reducing the required drug dose and minimizing systemic toxicity [127,128]. The feasibility of magnetothermally triggered drug release from Fe_3_O_4_@mSiO_2_ nanocarriers functionalized with thermoresponsive gatekeepers has been demonstrated in several studies.

### Current Preclinical and Clinical Evidence

Preclinical evidence for magnetothermal Fe_3_O_4_@mSiO_2_ drug-delivery systems remains dominated by in vitro release studies and cell models (Table 3). AMF-responsive release and enhanced cytotoxicity have been reported [102,108,127,128,130]. Still, quantitative comparison is hindered by incomplete reporting of iron-normalized SAR, AMF amplitude and frequency, nanoparticle concentration, achieved temperature or thermal dose, passive-leakage controls, and temperature-matched isothermal controls. In vivo evidence is limited. Guisasola et al. [109] reported tumor inhibition with a DOX-loaded carrier combined with AMF, but biodistribution, nanoparticle persistence, thermal dose, and long-term safety were not comprehensively characterized.

In practice, AMF conditions used in magnetic hyperthermia vary widely depending on the experimental setup and intended application. Many in vivo magnetic nanoparticle hyperthermia studies operate within the approximate frequency range of 100–500 kHz, although lower- and higher-frequency systems have also been reported [131].

Clinical experience with magnetic nanoparticle hyperthermia has been most prominently represented by the MagForce magnetic fluid hyperthermia platform, described as NanoTherm^®^ nanoparticles used with the MFH 300F applicator incorporating the NanoActivator^®^ F100 thermometry unit [132,133,134,135,136]. The applicator operated at a frequency of 100 kHz, while the magnetic-field amplitude was selected individually according to the measured nanoparticle distribution, predicted heat deposition, estimated tissue perfusion, treatment geometry, safety margins, and patient tolerability [132,136]. In the recurrent glioblastoma study, the field amplitude could be adjusted between 2 and 15 kA m^−1^ [136]. The applicator used in the early prostate studies was capable of generating field strengths of up to 18 kA m^−1^, although the field amplitudes actually tolerated and applied to patients were substantially lower [132,133,134]. The nanoparticle formulations used in both studies should be distinguished. In the 2011 glioblastoma study, NanoTherm^®^ AS1 was described as an aqueous dispersion containing 112 mg Fe mL^−1^, with magnetite (Fe_3_O_4_) cores approximately 12 nm in diameter and an aminosilane coating intended to stabilize the nanoparticle deposits within the treated tissue [136]. In the earlier prostate studies, the magnetic-fluid formulation was described as containing approximately 120 mg ferrite mL^−1^, with an average magnetic-core diameter of approximately 15 nm and an aminosilane-type coating [133,134]. The administered magnetic-fluid volume depended on tumor size, anatomical location, and the planned spatial distribution of the nanoparticle deposits [133,135,136]. In the prostate cancer case, approximately 12–12.5 mL of the formulation containing 120 mg ferrite mL^−1^ was administered, and the nanoparticle deposits remained detectable by computed tomography for at least six weeks, enabling repeated heating sessions without reinjection [133]. In the recurrent glioblastoma study, the median injected magnetic-fluid volume was 4.5 mL, corresponding to approximately 0.50 g Fe, and magnetic thermotherapy was combined with fractionated stereotactic radiotherapy rather than evaluated as an independent treatment. In this phase II study, 66 patients were enrolled, seven of whom did not fulfil the inclusion criteria; consequently, 59 patients with recurrent glioblastoma were included in the overall-survival analysis. The median overall survival from the diagnosis of the first tumor recurrence was 13.4 months, with a 95% confidence interval of 10.6–16.2 months. The authors classified the overall adverse effects as moderate and reported no serious complications, although headaches, seizures, motor disturbances, thermal stress, tachycardia, and alterations in blood pressure were observed during the treatment period [136].

Collectively, these clinical studies demonstrate the technical and clinical feasibility of generating localized hyperthermia using administered magnetic nanoparticles. They also emphasize the importance of patient-specific treatment planning, accounting for tumor volume and geometry, nanoparticle concentration and spatial distribution, tissue perfusion, magnetic-field amplitude, patient tolerability, exposure duration, thermal coverage, and the prevention of excessive heating in adjacent healthy tissues [132,133,134,135,136]. However, these studies primarily demonstrate the feasibility and tolerability of magnetic nanoparticle hyperthermia. Well-designed controlled comparative trials are still required to establish its independent oncological efficacy and any associated survival benefit.

Importantly, these clinical studies evaluated locally administered iron-oxide magnetic hyperthermia, not systemically administered thermoresponsive Fe_3_O_4_@mSiO_2_ drug-delivery carriers. No clinical trial has yet established the pharmacokinetics, controlled release, combination efficacy, or long-term safety of the complete platform considered in this review. The evidence chain therefore remains discontinuous: clinical feasibility exists for nanoparticle-mediated heating, while gated Fe_3_O_4_@mSiO_2_ chemotherapy remains preclinical. Claims of clinical readiness should make this distinction explicit.

## 7. Future Perspectives and Translational Challenges

Future progress in magnetothermally responsive Fe_3_O_4_@mSiO_2_ nanocarriers will depend less on adding further functionalities than on converting laboratory prototypes into reproducible and clinically usable products. The main priorities are standardized magnetothermal testing, scalable and sterile manufacturing, component-resolved pharmacokinetics and degradation, quantitative image-guided treatment, and prospective validation of computational design tools.

### 7.1. Standardization and Reproducibility

Future research on magnetothermally responsive Fe_3_O_4_@mSiO_2_ nanocarriers should move from the isolated optimization of individual components toward an integrated design strategy. Magnetic-core size, crystallinity, silica-shell thickness, pore architecture, polymer grafting density, and surface chemistry should be optimized simultaneously because improvements in SAR, drug loading, pore closure, colloidal stability, and release kinetics often involve competing trade-offs. Multi-objective experimental design, mechanistic modeling, and machine-learning approaches may help identify formulations that balance these properties more effectively.

A central priority is the standardization of magnetothermal testing. In an interlaboratory study involving 21 European laboratories, Wells et al. [105] reported good repeatability within individual laboratories but poor agreement between laboratories, with standard deviations of approximately ±30–40% in the ILP values. These findings demonstrate that systematic differences in instrumentation, experimental configuration, field calibration, and data analysis can substantially affect the reported heating performance.

The identified sources of variability included uncertainties in AMF amplitude, differences in coil design and field homogeneity, sample volume and positioning, thermal insulation and environmental heat transfer, temperature-probe type and positioning, sample stability, and the method used to analyze the temperature–time curves. Consequently, SAR or ILP values obtained using different instruments and protocols should not be compared without considering the corresponding experimental conditions and measurement uncertainties.

Future studies should therefore report the applied field amplitude and frequency, sample volume and position, coil and calorimeter configuration, temperature-probe type and position, sample concentration and stability, thermal boundary conditions, data-analysis method, and associated uncertainty. Repeated measurements, harmonized calorimetric protocols, interlaboratory comparisons, and the development of suitable reference or calibration materials are required to improve reproducibility and enable more meaningful comparisons between published studies [105].

Because heating performance measured in an aqueous suspension may not reflect that observed under physiological conditions, magnetothermal characterization should also be performed in biologically relevant media, viscosity-matched gels, immobilized states, and intracellular environments [24,25,137].

### 7.2. GMP Manufacturing, Sterilization, Scale-Up, Pharmacokinetics, and Regulation

Clinical development should begin with a target product profile defining the indication, administration route, dose, AMF exposure, release mechanism, and storage conditions. FDA guidance emphasizes that nanomaterial-containing products should be assessed according to their specific structure, composition, manufacturing process, stability, and biological behavior rather than being treated as a single regulatory class [138]. Recent European regulatory analysis similarly highlights manufacturing control, harmonized characterization, classification, and early interaction with regulators [139].

For Fe_3_O_4_@mSiO_2_ formulations, batch specifications should include iron-oxide phase and core size, shell thickness and porosity, hydrodynamic size and aggregation, iron/silicon/polymer/drug content, residual CTAB and solvents, endotoxin, sterility or bioburden, magnetic properties, SAR, passive leakage, triggered release, and stability. Acceptance ranges should be predefined, supported by orthogonal analytical methods, and related to biological and functional performance. Comparability should also be demonstrated after changes in scale, manufacturing site, raw materials, sterilization, or storage.

Sterilization must be incorporated during formulation development. Heat and irradiation can alter nanoparticle size, aggregation, surface stabilizers, and drug-release properties, whereas sterile filtration may be unsuitable for larger, aggregated, or highly viscous formulations [140]. The selected procedure should therefore be validated through pre/post-sterilization comparisons of physicochemical, magnetic, and release properties. Sterility assurance does not replace independent endotoxin control [138]. When terminal sterilization is incompatible with the formulation, validated aseptic processing may be required.

Scale-up must preserve precursor addition, mixing, phase transfer, heat and mass transfer, surfactant removal, washing, and functionalization. Gram-scale production of magnetic mesoporous silica nanoparticles [141] and continuous kilogram-scale production of mesoporous silica particles [142] have been demonstrated. Nevertheless, the former was not developed as a GMP drug product, whereas the latter did not include a magnetic core, therapeutic cargo, or responsive coating. These studies are therefore encouraging but do not establish scalable GMP manufacture of a complete Fe_3_O_4_@mSiO_2_ nanomedicine.

Pharmacokinetic studies should distinguish the intact nanocarrier, magnetic core, silica shell, coating, encapsulated drug, and released drug. Measurements of total iron or drug alone cannot determine carrier integrity or degradation. When feasible, orthogonal analytical methods or independent stable labels should be employed to follow the individual components and their degradation products [138].

Because treatment also requires an AMF applicator, development may follow a drug–device combination–product framework, depending on the jurisdiction, primary mode of action, and final product presentation [139,143]. Applicator calibration, field homogeneity, thermometry, dosimetry, exposure limits, electromagnetic compatibility, and fail-safe controls should consequently be developed together with the nanocarrier.

### 7.3. Biodegradable and Clinically Translatable Platforms

Future formulations should remain stable during treatment but subsequently degrade into identifiable and safely metabolized or excreted products. Potential strategies include post-synthetic control of mesoporous-shell porosity [144], and based on related non-magnetic platforms, the incorporation of redox-cleavable organosilica frameworks [145]. However, the integration and degradation of such frameworks in complete magnetothermal Fe_3_O_4_@mSiO_2_ nanocarriers remain to be demonstrated. Faster degradation may also increase premature drug leakage, expose the magnetic core, or alter heating during repeated treatments; these trade-offs must therefore be evaluated experimentally.

Direct evidence shows that the extracellular and intracellular dissolution rates of magnetic-core mesoporous-silica-shell nanoparticles can be modified through post-synthetic control of shell porosity. Intracellular silica dissolution was substantially slower than extracellular dissolution and depended on the nanoparticle concentration within intracellular vesicles [144]. Evidence obtained with PEGylated ultrasmall iron-oxide nanoparticles, rather than complete Fe_3_O_4_@mSiO_2_ carriers, further indicates that iron-oxide degradation and metabolism depend on dose, organ, and host iron status [146].

Biodegradability should therefore be demonstrated by time-resolved identification of degradation products, component-specific mass balance, retention and clearance, toxicology, and the preservation or loss of heating and release functions over time, rather than inferred solely from the known fate of soluble silicate or endogenous iron. From a translational perspective, a simpler carrier that achieves adequate heating and controlled release may be preferable to a highly multifunctional platform that is difficult to manufacture, characterize, and qualify reproducibly.

### 7.4. Multimodal, Image-Guided, and Personalized Theranostics

Evidence from related iron-oxide and magnetic nanoplatforms illustrates the opportunities for integrating imaging, thermometry, and spatially controlled heating. MRI-tracked magnetic hyperthermia has recently been demonstrated in a preclinical glioblastoma model [147], while MPI has enabled quantitative tracer imaging and spatially localized heating in vivo [148]. An integrated MPI–hyperthermia–MPI-thermometry platform has also been reported, although its evaluation was primarily performed in phantoms and still requires in vivo validation [149]. Heater–thermometer nanoplatforms with operando temperature monitoring provide further proof-of-concept, but do not yet constitute clinically validated closed-loop systems [150].

The next step is to establish calibrated relationships among imaging signal, local iron concentration, heat generation, temperature, thermal dose, and drug release. Personalized treatment planning could combine anatomical imaging, nanoparticle distribution, perfusion, field maps, and bioheat-transfer models. Closed-loop systems could then adjust AMF power, heating location, and exposure duration using thermal-dose feedback. Clinically useful implementations will require spatial calibration, real-time uncertainty estimates, motion and perfusion correction, predefined fail-safe limits, and validation in heterogeneous large-animal or human-scale geometries.

Multimodal functions should be retained only when they improve treatment decisions, dosimetry, or safety. Otherwise, additional imaging labels, targeting ligands, and sensors increase formulation complexity, dose, analytical burden, and regulatory uncertainty without demonstrated clinical benefit.

### 7.5. Computational Optimization and Artificial Intelligence

Computational modeling and artificial intelligence can support multi-objective optimization of synthesis, heating, pharmacokinetics, and drug release. A recent machine-learning study compiled 1850 SAR measurements from 84 publications and evaluated 30 physicochemical and experimental descriptors. AMF amplitude and frequency, nanoparticle concentration, and magnetic-core surface area were identified among the most influential predictors of SAR [106]. However, prediction variability increased for particles outside the best-represented regions of the training dataset, demonstrating the limitations of extrapolating literature-trained models.

A broader machine-learning analysis of 745 preclinical studies similarly demonstrated the potential of data-driven design for inorganic cancer nanomedicine and emphasized the need for more comprehensive databases and standardized reporting [110]. To the best of our knowledge, end-to-end AI-guided design, drug delivery, and treatment adaptation have not yet been demonstrated for Fe_3_O_4_@mSiO_2_ nanocarriers. These approaches should therefore be presented as emerging opportunities rather than established translational solutions.

Near-term applications should focus on the design of experiments, prediction of synthesis–structure relationships, SAR and release optimization, identification of critical manufacturing variables, and integration of imaging with pharmacokinetic and thermal models. Datasets should include complete AMF conditions, normalization basis, aggregation state, uncertainty, and negative results. Models should define their applicability domain and be prospectively validated using independently manufactured formulations.

Physics-informed digital twins may eventually support patient-specific treatment planning by integrating field distribution, nanoparticle transport, magnetic relaxation, perfusion, heat transfer, degradation, and drug-release kinetics. However, their predictions should remain subordinate to calibrated measurements and prospective biological validation.

## 8. Conclusions

Fe_3_O_4_@mSiO_2_ nanocarriers provide an attractive framework for combining magnetic heating, mesoporous drug storage, and thermoresponsive release within a single platform. However, the available evidence does not yet establish the clinical readiness of the complete system. A central challenge is not only the independent demonstration of each function, but their simultaneous preservation in the final formulation and under biologically relevant conditions. Silica and polymer incorporation dilute the magnetic fraction; aggregation and immobilization can restrict Brownian relaxation; thicker shells can increase loading while reducing heating per unit carrier mass; and dense polymer grafting can improve pore closure while limiting pore accessibility and altering the apparent LCST.

Future magnetic cores should therefore retain effective Néel- or dynamic-hysteresis-mediated heating when whole-particle rotation is restricted in viscous media, intracellular compartments, or tissue. At the same time, thermoresponsive gatekeepers must maintain stable pore closure at 37 °C in serum-containing media and undergo rapid, complete, and reversible conformational collapse at approximately 40–45 °C, thereby increasing pore accessibility and enabling controlled drug release. These properties should be evaluated in the complete drug-loaded carrier because LCST, aggregation, pore accessibility, SAR, and release kinetics may change after each functionalization step.

Meaningful progress also requires harmonized magnetothermal and release testing. SAR and ILP should be reported together with field amplitude, frequency, H × f, iron and carrier concentrations, sample volume and geometry, medium viscosity, aggregation state, normalization basis, temperature-measurement method, and uncertainty. Testing should progress from water to physiological media, immobilized models, cells, and tissues.

Biological validation should extend beyond short-term viability and tumor-growth measurements. Component-specific pharmacokinetics, biodistribution, degradation, long-term retention, and excretion of the magnetic core, silica shell, polymer coating, and cargo are required, together with hemocompatibility, immunogenicity, oxidative-stress, repeated-dose, and chronic-toxicity studies. Importantly, current clinical evidence demonstrates the feasibility of locally administered iron-oxide hyperthermia but not the clinical efficacy or safety of systemically administered, thermoresponsive Fe_3_O_4_@mSiO_2_ drug-delivery carriers.

Clinical translation will ultimately depend on reproducible scale-up, validated sterilization and endotoxin control, batch specifications linked to biological performance, storage stability, and integrated development of the nanocarrier and AMF applicator as a potential combination product. Image-guided feedback, computational optimization, and artificial intelligence may improve treatment planning and multi-objective formulation design, but their value must be established prospectively using standardized and independently generated data. Rather than maximizing multifunctionality, future development should prioritize the simplest architecture capable of achieving reproducible heating, minimal premature drug leakage, controlled release, measurable therapeutic benefit, and safe clearance.

## Figures and Tables

**Table 1:** Comparison of Fe_3_O_4_ core-synthesis routes and their effects on critical magnetic attributes.

Route	Main Advantages	Main Limitations	Typical Influence on Core Properties	Ref.
Co-precipitation	Aqueous; simple; short reaction time; low cost; high yield; potentially scalable	Limited size and shape control; sensitive to pH, Fe^2+^/Fe^3+^ ratio, base, mixing and oxidation conditions; batch variability	Commonly produces quasi-spherical particles, although morphology can be tuned through reaction conditions; broad size distribution; crystallinity and magnetic properties are strongly process-dependent	[36,37,38,39,40,41]
Thermal decomposition	Narrow size distribution; high crystallinity; tunable size and morphology; high reproducibility	High reaction temperatures; high cost; organic solvents; hydrophobic surfaces commonly require ligand exchange or phase transfer	Generally produces monodisperse, highly crystalline particles with tunable dimensions and potentially high saturation magnetization; phase purity and magnetic properties depend strongly on precursor quality, solvent, surfactant concentration, heating rate, temperature and reaction time	[36,37,39,42,43,44]
Hydrothermal/solvothermal	Good crystallinity; broad control over size and morphology; possibility of producing spheres, cubes, rods, hollow particles, and multicore structures	High temperature and pressure; long reaction times; sealed reactors required; scale-up and heat-transfer control may be difficult	Can produce crystalline particles and polycrystalline assemblies over a broad size range. Phase purity, crystallite size, morphology, aggregation and magnetization depend strongly on the solvent, additives, precursor concentration, temperature and reaction time	[36,37,38,45,46]
Polyol	Polyol can act as a solvent, reducing agent, and surface stabilizer; good control of growth, morphology, and aggregation; potential for scale production	High temperatures; relatively long reaction time; sensitive to polyol nature	Produces crystalline single-core particles or multicore clusters with tunable size, morphology, magnetic properties, and surface functionality	[36,37,39,47,48]
Microemulsion	Nanoscale droplets act as confined reactors; good control over nucleation and particle size; narrow size distribution; good dispersibility	Low yield; expensive; residual surfactants; complex washing; difficult scale-up	Commonly produces small, relatively uniform and superparamagnetic particles; particle size and magnetic properties depend on the microemulsion composition, water-to-surfactant ratio, and reaction conditions	[36,37,38,39,49]
Microwave-assisted	Rapid and relatively homogeneous heating; short reaction times; improved throughput	Requires specialized equipment; scale-up depends on reactor geometry and microwave-field homogeneity	Can rapidly produce relatively uniform, well-defined crystalline or semicrystalline particles; size, phase and magnetic properties depend on the reaction medium and microwave conditions	[37,38,39,50,51,52]

**Table 2:** Integrated design parameters, trade-offs, and characterization techniques for Fe_3_O_4_@mSiO_2_ magnetothermal nanocarriers.

Design Element	Key Parameters	Desired Function	Main Trade-Off	Characterization Techniques to Validate
Fe_3_O_4_ magnetic core	Core diameter; morphology; crystallinity; iron oxide phase; aggregation state; Fe content; saturation magnetization; coercivity; remanence; hydrodynamic diameter; polydispersity; surface charge; AMF responsiveness	AMF-induced heating; magnetic responsiveness; magnetic guidance; potential MRI contrast; colloidal stability	Heating efficiency and SAR may decrease after silica coating, particle aggregation, or dispersion in viscous or biological media; stronger AMF conditions may enhance heating but increase the risk of nonspecific tissue heating	TEM/HRTEM; SEM; DLS; zeta potential; XRD; Raman; Mössbauer/XPS; VSM; SQUID; AMF calorimetry (SAR)
mSiO_2_ shell	Shell thickness; pore size distribution; pore volume; specific surface area; silanol density; pore accessibility; structural integrity; surface functional groups; residual CTAB or other template; drug–silica affinity; loading capacity; encapsulation efficiency	Mesoporous drug reservoir; cargo protection; high drug loading capacity; cargo retention; controlled release; platform for polymer grafting; targeting; and surface functionalization	Thicker or highly porous shells may increase drug loading but also increase hydrodynamic size and dilute the magnetic response; silanol groups or incomplete template removal may reduce pore accessibility, alter colloidal behavior, or increase cytotoxicity	TEM/HRTEM; N_2_ adsorption–desorption; BET; BJH; DLS; zeta potential; FTIR; XPS; TGA; elemental analysis
Thermoresponsive gatekeeper	Polymer identity; LCST; grafting density; grafted-polymer amount; chain length; polymer architecture; anchoring chemistry; hydrophilicity; reversibility; temperature-dependent size and turbidity; passive leakage; AMF-triggered release temperature	Effective pore blocking and minimal drug leakage at 37 °C; reversible pore opening and controlled drug release under mild hyperthermia, typically at 40–45 °C; remote activation by AMF-induced heating	Dense grafting may improve pore closure but reduce pore accessibility, slow drug release, or shift the apparent LCST; insufficient grafting may cause premature leakage; LCST and release behavior may change in PBS, serum, or other biological media	FTIR; TGA; XPS; DLS vs. temperature; DSC; turbidimetry; UV–Vis spectroscopy; fluorescence spectroscopy; or HPLC

**Table 3:** Representative magnetothermally responsive Fe_3_O_4_@mSiO_2_-based nanocarriers for combined chemotherapy and magnetic hyperthermia. Abbreviations and symbols: alternating magnetic field (AMF); combination index (CI); doxorubicin (DOX); encapsulation efficiency (EE); folic acid (FA); gemcitabine (GEM); glutathione (GSH); hydrodynamic diameter (HD); product of magnetic-field amplitude and frequency (H × f); hydroxypropyl cellulose (HPC); loading capacity (LC); magnetic mesoporous silica nanoparticle (MMSN); mesoporous silica (mSiO_2_); saturation magnetization (M_s_); not reported (NR); poly(ethylene oxide-co-L-lactide) [P(EO-co-LLA)]; poly(N-isopropylacrylamide-co-methacrylic acid) [P(NIPAM-co-MAA)]; specific absorption rate (SAR); Brunauer–Emmett–Teller specific surface area (S_BET_); disulfide bond or disulfide-containing linker (SS); transmission electron microscopy (TEM); thermoresponsive polymer (TRP); and ultrasmall superparamagnetic iron oxide (USPIO).

System/Model	Size/Shell	Magnetic Properties	AMF/SAR Conditions	Loading/Encapsulation	Release and Outcome	Ref.
DOX–Fe_3_O_4_@mSiO_2_–P(EO-co-LLA); HeLa	TEM: ≈65 nm; Fe_3_O_4_ core ≈ 20 nm; mSiO_2_ shell ≈ 20 nm; HD ≈ 103–135 nm depending on polymer molecular weight)	Superparamagnetic; M_s_ = 48.7–59.5 emu g^−1^ for the polymer-coated formulations; SAR NR	250 kHz; field strength and particle concentration NR; ≈45 °C in 20 min and 47 °C in 30 min	LC ≈ 5.9–6.8 wt%; EE NR	Continuous AMF increased DOX release to ≈89.4% in 12 h. DOX-loaded carrier induced ≈71.5% HeLa-cell apoptosis without AMF and ≈93.7% with AMF; in vitro only	[102]
DOX-loaded Fe_3_O_4_/mSiO_2_ nanoparticles; HeLa	≈150 nm; several 15–20 nm Fe_3_O_4_ cores embedded in each silica matrix; shell thickness NR	Superparamagnetic; M_s_ = 4.2, 5.8 and 8.1 emu g^−1^ for formulations containing increasing Fe_3_O_4_ amounts; representative SAR = 9.1 W g^−1^ for Fe_3_O_4_/mSiO_2_-3 at 409 kHz and 150 G	238 or 409 kHz; 90–180 G; 50 mg mL^−1^ in water; 20 min. At 409 kHz and 150 G, Fe_3_O_4_/mSiO_2_-3 produced ΔT ≈ 43.9 °C	LC and EE NR	Sustained DOX release at pH 5.0 but very slow release at pH 7.4; low carrier cytotoxicity and cellular uptake demonstrated. Heating and release were evaluated separately, without combined therapeutic efficacy	[130]
DOX–MMSN@P(NIPAM-co-MAA); HeLa	≈255 ± 28 nm; Fe_3_O_4_ domains ≈ 15–20 nm; pore diameter ≈ 2.6 nm	Superparamagnetic; M_s_ = 6.2 emu g^−1^; SAR NR	409 kHz; up to 180 G (18 mT); ≈64.2 °C after 15 min at 180 G	LC = 38.6 μg mg^−1^ (≈3.86 wt%); EE NR	Low basal release at pH 7.4; release increased under acidic conditions, elevated temperature, and AMF. Combined treatment reduced HeLa cells viability to ≈23%, vs. ≈76% with DOX and ≈42% with AMF alone; in vitro only	[128]
DOX–MMSN@TRP; EL4 lymphoma allografts in C57BL/6 mice; intratumoral administration	Final HD ≈ 160 nm; MMSN precursor: ≈1511 m^2^ g^−1^ and pore volume ≈ 2.24 cm^3^ g^−1^; shell thickness NR	Iron content ≈ 95.5 μg mg^−1^; SAR = 178.5 W g^−1^ Fe	105 kHz; 18 kA m^−1^; H × f ≈ 1.89 × 10^9^ A m^−1^ s^−1^; 30 min on the injection day and two consecutive days; 1 mg carrier/tumor, corresponding to ≈182 μg Fe/tumor	LC = 25 μg mg^−1^ (2.5 wt%); EE NR	Only DOX–MMSN@TRP + AMF significantly inhibited tumor growth 48 h after the final exposure (*p* < 0.001); no macroscopic tumor-temperature increase was required. Short follow-up and no pharmacokinetic or biodistribution analysis	[109]
DOX/MMSN-SS-FA; HeLa and tumor-bearing mice	Fe_3_O_4_ cores ≈15 nm; S_BET_ ≈ 658 m^2^ g^−1^; total particle size and silica-shell thickness NR	and SAR NR	Frequency and field strength NR; 1 mg mL^−1^ suspension heated from 37 to ≈45 °C under AMF	LC ≈15 wt%; EE NR	At 24 h, ≈18% release at pH 7.4 and ≈39% at pH 5.0; additional redox- and AMF-responsive release. DOX/MMSN-SS-FA + AMF showed synergistic HeLa cytotoxicity (CI = 0.276) and ≈84% tumor-growth inhibition in vivo	[127]
Gemcitabine–HPC–porous-silica-coated USPIO clusters; PANC-1 cells and xenografts	Individual USPIOs ≈ 7 ± 1 nm; cluster core ≈ 59 ± 6 nm; silica shell ≈ 18 ± 2 nm; pores ≈ 5 nm; =113.5 m^2^ g^−1^	Superparamagnetic; M_s_ = 61 emu g^−1^ for USPIO clusters and M_s_ = 20 emu g^−1^ for the final carrier; SAR = 183 W g^−1^	199 kHz; 175 Oe (13.9 kA m^−1^); 1 mg mL^−1^; ≈45 °C within 100 s; in vivo exposure for 30 min	Loading efficiency ≈ 66% from a 20 wt% GEM feed; LC not independently reported	≈37% GEM release at 45 °C vs. ≈8% at 37 °C after 180 min. PANC-1 viability was ≈21% at 42 °C and ≈12% at 45 °C. In vivo, chemohyperthermia produced ≈38% apoptosis vs. 17.5% with chemotherapy and 14.7% with hyperthermia alone	[108]

## Data Availability

No new data were created in this study. Data sharing does not apply to this article.

## Abbreviations

The following abbreviations are used in this manuscript:

AA	Acrylic acid
AC	Alternating current
AI	Artificial intelligence
AMF	Alternating magnetic field
APTT	Activated partial thromboplastin time
AS1	NanoTherm^®^ AS1 formulation
BET	Brunauer–Emmett–Teller
BJH	Barrett–Joyner–Halenda
CI	Combination index
CTAB	Cetyltrimethylammonium bromide
DLS	Dynamic light scattering
DMPC	1,2-Dimyristoyl-sn-glycero-3-phosphocholine
DNA	Deoxyribonucleic acid
DOX	Doxorubicin
DSC	Differential scanning calorimetry
EE	Encapsulation efficiency
ELPs	Elastin-like polypeptides
F100	NanoActivator^®^ F100 system
FA	Folic acid
FDA	U.S. Food and Drug Administration
Fe_3_O_4_@mSiO_2_	Magnetite-core/mesoporous-silica-shell nanoparticle
FTIR	Fourier transform infrared spectroscopy
GEM	Gemcitabine
GMP	Good manufacturing practice
GSH	Glutathione
HD	Hydrodynamic diameter
HPC	Hydroxypropyl cellulose
HPLC	High-performance liquid chromatography
HRTEM	High-resolution transmission electron microscopy
ILP	Intrinsic loss power
LC	Loading capacity
LCST	Lower critical solution temperature
MAA	Methacrylic acid
MFH	Magnetic fluid hyperthermia
MMSN/MMSNs	Magnetic mesoporous silica nanoparticle(s)
MPI	Magnetic particle imaging
MRI	Magnetic resonance imaging
MSN/MSNs	Mesoporous silica nanoparticle(s)
MPS	3-Methacryloxypropyltrimethoxysilane
*M_s_*	Saturation magnetization
NIPAM/NIPAAm	N-Isopropylacrylamide
NR	Not reported
PAAm	Polyacrylamide
PBS	Phosphate-buffered saline
PDAC	Pancreatic ductal adenocarcinoma
PEG	Polyethylene glycol
PEI	Polyethylenimine
P(EO-co-LLA)	Poly(ethylene oxide-co-L-lactide)
PET	Positron emission tomography
P(GME-co-GEE)	Poly(glycidyl methyl ether-co-glycidyl ethyl ether)
P(NIPAM-co-AA)	Poly(N-isopropylacrylamide-co-acrylic acid)
P(NIPAM-co-MAA)	Poly(N-isopropylacrylamide-co-methacrylic acid)
P(NIPAM-co-MPS)	Poly(N-isopropylacrylamide-co-3-methacryloxypropyltrimethoxysilane)
PNIPAM	Poly(N-isopropylacrylamide)
PNVCL	Poly(N-vinylcaprolactam)
POEGMA	Poly(oligo(ethylene glycol) methyl ether methacrylate)
PPG	Polypropylene glycol
PVP	Polyvinylpyrrolidone
ROS	Reactive oxygen species
SAR	Specific absorption rate
SEM	Scanning electron microscopy
SPION/SPIONs	Superparamagnetic iron oxide nanoparticle(s)
SQUID	Superconducting quantum interference device
SS	Disulfide bond or disulfide-containing linker
TAB004	Anti-tMUC1 monoclonal antibody
TEOS	Tetraethyl orthosilicate
TEM	Transmission electron microscopy
TGA	Thermogravimetric analysis
tMUC1	Tumor-associated mucin 1
TRP	Thermoresponsive polymer
USPIO/USPIOs	Ultrasmall superparamagnetic iron oxide nanoparticle(s)
UV–Vis	Ultraviolet–visible spectroscopy
VSM	Vibrating sample magnetometry
XPS	X-ray photoelectron spectroscopy
XRD	X-ray diffraction
